# Medical and Philosophical Intelligence

**Published:** 1817-05

**Authors:** 


					MEDICAL and PHILOSOPHICAL INTELLIGENCE.
To the Editors of the London Medical and Physical Journal.
GENTLEMEN,
IN your last Journal, annexed to " more last words" of Mr.
Woodham, you very properly recommend that the altercation
between that gentleman and myself should cease, the merits of the
case being sufficiently before the public. This, as it must appear
by a postscript affixed to my last communication, I had already
resolved on, feeling myself then, as I do now, perfectly at ease
jespccting it.
Mr. Woodham, however, still restless, seems to li? extremely
anxious to have the last word, as it is ordinarily called, conceiving,
probably, that that determines the victor.
In order, therefore, to put an end to this species of contention,
which is the only circumstance remaining unsettled between us, I
shall take the liberty of communicating my idea of the last word
in a literary contest, by the following quotation:
" Quoth Cibber to Pope, though in verse you foreclose,
I'll have the last word, for by G-d I'll write prose;
Poor Colley! thy reas'ning is none of the strongest,
For know,?the last word is the word that lasts longest."
Thus it happens, I trust, that Mr. Woodham and myself are
each accommodated in his own way.
I am, Gentlemen,
Your much obliged and obedient Servant,
Oxford; Aprils, 1817. Richakd Walker.
[We now trust both these gentlemen will be as well satisfied at
the conclusion of this innocent controversy as our readers must be.
Pur Nestoreaa correspondent seems to acknowledge his failing at
* Jong
Medical and Philosophical Intelligence. 425
long words, long sentences, and long letters and contents for $
finale lengthened out in his own way.?Edit.]
To the Editors of the London Medical and Physical Journal.
GENTLEMEN,
I accede to your recommendation, and, should I be so fortunate
as to be considered, by the public, as the man of superior judg-
ment, I shall not feel any spleen, though the qualities of wit and
humor be awarded to Mr. Walker.
I am, gentlemen,
London ; Your most obedient servant,
April 25, 1817. James Woodiiam*
To the Editors of the London Medical and Physical Journal*
GENTLEMEN,
I herewith send you the following list* the result of two
days' Botanical Excursions; and, if any of your readers have the
curiosity to refer to our first labors last spring, it will be found*
that the difference of the two seasons is really surprising. Early
springs are in general considered as unfavorable to crops in this
country; but, the long series of dry weather has rendered the corn
in general very fine. The wheat near London is excessively
strong, and, if the weather should continue favorable for its
growth hereafter, we may anticipate, with the blessing of Pro-
vidence, a plentiful harvest. The lent corn, in some places, is
come up, and appears very fine. Clovers are also very luxuriant;
and the early crops of grass look very healthy. Should favor-
able rains fall during the month of May, the hay harvest cannot
fail to be very productive.
On the 10th, we visited Hampstead Heath, Bishop's Wood, and
Ilighgate Archway.
Veronica arvensis
Juncus pilosus
campestris
? sjlvaticus
Ranunculus hecteraccus
Viola canina
Anemone nemerosft
Salix fusca
Equisetum palustre
Leontodon taraxicuiti
Oxalis Acetosella
Prunus spinosa
Ajuga reptans
Vaccinum Myrtillus
Salix Caprnea
Euphorbia Amygdaloidq*
Primuli acaulis
Fragaria vesca
Salix amygdaloide*
Arenaria rubra
Ranunculus bulbosus
Geranium inolle
Veronica serpyllifolia.
Eqnisetum arvense
Fragaria steriiis
Ranunculus auricomus
Scilla nutans
Primula officinalis
Spartium scoparium
Cardamine ainara
Pinus sjlvestris
Brassica Rapa
Ranunculus hirsutus
Anthoxanthus adoratum
Stellaiia Holostea
Ranunculus aquatilis
Prunus Cerasus
Salix rubra
Allium ursinum
so*. 219* 1
426 Medical and Philosophical Intelligence?
On Thursday the 17th instant, Battersea Fields.
Carex acuta
Lithospermum arvense
Euphorbia Helioscopia
Viola bicolor
Scandix Pecten veneris
Carex riparia
Callitriche verna
Ch?raphyHum sylvestre
Equisetum liniosum
Veronica Chamsedrys
Anthenais arvensis
Scandix Anthriscus
Cerastium viscosum
Lamium dissectum
Erysimum officinale
Thlaspi arvense
Fumaria capreoiata
Tussilago Petasites
Plantago lanceolata
My next excursions will be on Thursday the 8th of May, when
I shall meet at the [led House, Battersea; on Thursday the 15th,
at the Castle, Hampstead Heath; and, on Thursday the 22d, at
the French Horn, at Charlton. The times of meeting at each
place, is to be at ten in the forenoon.?If the mornings prove wet,
the excursion will be put off till a future day, which will be ar-
ranged at the following Monday's lecture.
I shall begin a fresh Course of Lectures on Botany, on Monday
the 5th of May, in the Library at the Botanic Garden, Sloane
Street, at three o'clock in the afternoon. 1
I am, most respectfully, gentlemen,
Your very obedient servant,
Wm. Salisbury.
To the Editors of the London Medical and Physical Journal.
GENTLEMEN,
In your Journal, No. 217? your correspondent, Mr. William
Cooke, recommends a solution of the muriate of soda instead of
spirit, for the preservation of anatomical preparations. The spe-
cific gravity of that solution is a serious objection to its use; and
its want of transparency prevents any delicate structure being
well displayed. These objections are not made theoretically, but
are the result of practical observations, as it was recommended by
the late Thomas Young, surgeon, and tried by George Young and
John Taunton, many years since. Even in the preservation of
large parts, such as were used only for occasional demonstration,
spirit was found to be much better than this.?Your remarks,
Mr. Editor, will oblige A Correspondent.
[The muriate of soda uged on this occasion was not the salt of
commerce, but that in large transparent crystals.?Edit.]
Bath Literary and Philosophical Society.?A paper by Dr.
Wilkinson, o? the Rise of Fluids in capillary Tubes was after-
wards read. Its object was to show, that the experiments on this
subject do not accord with the theoretic calculations of Professor
Atwood of Cambridge, and other philosophers ;?that the results
do not correspond with any fixed rule, but are entirely dependent
on certain conditions of the tube; and that no dependence can be
' placed
Medical and Philosophical Intelligence. 427
placed on any deductions from mathematical investigations as to
?what has been termed capillary attraction.?On this subject, we
have been for some time making experiments, the result of which is,
that the supposed filtration by capillary attraction will extend no
further than the syphon; that is, that the filaments on the outside
of the vessel must be brought lower than the fluid within.
Dr. Spurzheim's Second Demonstration of the recent Brain, as
we remarked in our last, took place at the Anatomical Theatre of
St. Bartholomew's. The lecturer's principal object was to demon,
strate those facts which had been disputed or contradicted by his
adversaries. He showed, for instance, that there is brown matter
in the interior of the cerebellum, in the pons varolii; and that
there is no part of the brain in which the same may not be disco-
vered. The decussation of the anterior pyramids was carefully
pointed out, and the structure of cerebellum, more particularly the
variety of appearances which the corpus dentatum exhibits, ac-
cording to the manner in which it is cut or examined. It should
be remarked, that Dr. S. begins his examination at the base of the
brain, and demonstrates the different parts in their connexion
without slicing and destroying them. The pons varolii, and most
of those parts which have acquired a number of whimsical names,
he distinguishes as origins and commissures of the various parts of
the brain. This very much assists thfe memory, by a perpetual
appeal to the judgment, instead of requiring the constant recol-
lection of an unsatisfactory nomenclature. The transverse fibres
of the pons are considered as the means of uniting the two hemi-
spheres of the cerebellum, while the longitudinal bundles of the
pons bring the parts of the brain proper, into communication
with the medulla oblongata. In the corpus callosum he demon,
strates the fibres running sideways in a most beautiful and satis,
factory manner, by gently scraping with the scalpel: showing, at
the same time, that a longitudinal scrape exhibits only a smooth
appearance. The corpus callosum proves the commissure or
uniting medium of the two hemispheres and their convolutions.
Some of these were unfolded with the greatest delicacy, to show
the manner in which that organ is disposed so as to occupy the
least possible space with the largest surface. So delicate is the
structure of the whole, that it would hate been impossible not to
suspect some slight of hand in the division of the white substance
into two layers, had not the lecturer had recourse to the blow-
pipe, the force of the air from which was sufficient to exhibit the
natural division in the most satisfactory manner. This led to the
explanation of those causes by which an hydrocephalic subject re-
tains his senses, his powers of reflexion, his affections, and facul-
ties. By the slow manner in which the water increases, the layers
are divided; and, if the water still increase, the fibres gradually
are directed in an horizontal instead of a vertical direction. Thus
the brain retains its full bulk, as has been proved by weight,
3 i 2 w here
428 Medical and Philosophical Intelligence.
?where it has been expanded so thinly round the enlarged cranium
as to have been suspected to be altogether, or nearly all, absorbed.
"With much candour Dr. S. informed his hearers that the existence
of the brain in hydrocephalic heads had been noticed by the accu-
rate Morgagni, who blames some of "his predecessors and contem-
poraries for having overlooked the brain in its expanded state.
Dr. S. assures us, that, however thin the brain may appear, or
even though it may be thought to have disappeared altogether, yet
that in one such case he had weighed it against the brain of sub-
jects of the same age, and found no difference.
The following account of the examination of the cranial con-
tents, in two subjects approaching to icliotcy, are worth recording;
and we hope will prove the means of accumulating other facts in
illustration of the properties of this important organ.?These are
from the " Repository.''
A man, aged 60, approaching the condition of an f< idiot, tall,
and very thin, had been affected for three years with giddiness and
pain in the head, which had from time to time been relieved by
local bleeding, blisters, and setons to the neck ; but most by a
caustic applied at the point where the coronal and sagittal sutures
meet. He always had, combined with this affection of his head,
more or less of griping with slimy stools; which latter symptoms,
for the last twelve months, had been particularly severe, producing
an almost constant diarrhoea and tenesmus.
* * * * *
" Sectio cadaveris, eighteen hours after death.?Head. Nothing
remarkable was observed in the scalp or dura mater. On re-
moving the latter, the tunica arachnoidea was found thickened and
opake, and, on puncturing it, a serous fluid, to the amount of
about three ounces, escaped from between it and the pia mater;
Some of the vessels of the pia mater were nearly empty, and ap-
peared to be larger than common. There was about an ounce of
serous fluid in the lateral ventricles. Both the cineritious and
medullary substance of the brain, when cut intp, had a more vas-
cular appearance than in the natural state of that organ. The ob-
ject the most curious was an anomaly which prevailed in the struc-
ture of the brain: the posterior cornua of the ventricles were ab-
solutely wanting, so that there was no trace of the hippocampus
minor. This deviation did not appear to be the effect of disease;
for the structure of the brain did not differ in any other respect
from that disposition of parts which is common in other brains.''
" The singularity in the construction of the brain in the above
case, with its attendant weakness of intellect, struck me so forcibly
that I determined to embrace every opportunity that offered of
minutely examining the cranium of every patient where there was
an approach to idiotism.
<? It was not long before a man, aged twenty-five, and who was
little superior in understanding, died from erysipelatous inflam-
mation attacking the head. Upon examining the structure of the
lateral
Medical and Philosophical Intelligence. 429
lateral vcntrlcles, the anterior aud interior cornua appeared fully-
developed ; but here, as in the first case, the posterior cornua
were wholly deficient."
These histories may make part of future records. Though the
surgeon discovered 110 other difference in the two brains than the
want of posterior cornua, yet, when we consider the very imper-
fect manner in which the demonstration of the brain has been hi.
therto conducted in our best anatomical schools, we may, without
breech of candour, presume that a Gallite would have perceived
other peculiarities. Dr. Spurzheim assures us, that though, for the
most part, every brain consists of the same exterior general form,
yet, in marking minutely the outlines, there is found as great a
variety as in the various features of the human face. He finds no
? two brains exactly alike, any more than two faces.
All Prof. Davy's recent experiments with damaged flower seem to
mark carbonate of magnesia as the proper remedy. The quantity-
used is from 20 to 30 grains to ftj. of flower.
One of those trials has lately taken place for murder which so often
astonish ug, by proving how little attention is paid to the most im-
portant phenomenon of the stomach, in cases which are decided
concerning the life of a suspected person.
The prisoner, Mr. Robert Saul Donnall, a surgeon, practising
at Falmouth, was indicted for no less a crime than that of destroy-
ing his mother, by giving her arsenic with her food, at his own
house. Dr. Edwards was consulted during her short illness, and
gave the following deposition :
" Dr. Richard Edwards gave evidence, that he had been sixteen
years in the profession. When called in between four and five
o'clock on the morning of the 4th of November, he asked the
prisoner some questions respecting Mrs. Downing: the prisoner
said that she had an attack of the cholera morbus; and that she
had been similarly affected about a fortnight before. The witness
went into her room, and found that she required a little rousing
before she could answer any questions : she complained of great
heat, and her pulse was frequent and fluttering: she had 110 sick-
ness at that time, but was in very great danger. He understood,
from the prisoner, that he had given her an opening medicine, an
emetic, a saline draught, and some opium. The witness wrote a
prescription, with a view to remove something offensive which ap,
peared to be in her stomach or bowels. He had never met with a
case of cholera morbus that had produced death in a shorter time
than three or four days; and he was of opinion that that disorder
was not the cause of the death of Mrs. Downing. On Thursday
the 7th of November, the witness was requested to go and exa-
mine the body, by opening it, having previously heard of the sus-
picion of poison. He saw the prisoner at the ^ouse, and a surgeon
of the name of John Street. After taking the body out of the
shelly
430 Medical and Philosophical Intelligence?
?hell, the witness perceived that the prisoner was tucking up his
sleeves and preparing to open the body; but the witness told him
that he must have nothing to do with the operation. On opening
the stomach, the contents were poured into a basin, and, after the
lapse of a few minutes, they were examined, but no deposit of
any thing heavy or solid could be discovered. The stomach was
in a state of stellated inflammation, and both the coats of the sto.
mach were softened, as if by the action of some corrosive sub-
stance ; one of them could be scraped off by the finger nail. Th^
blood-vessels of the stomach were more than ordinarily turgid,
but the liver and lungs were sound : the heart the witness did not
examine. The basin with the contents of the stomach had been
carefully placed on a chair; witness observed at the time, aloud,
that Ihey must be preserved, and the prisoner must have heard it.
They then proceeded to inspect the intestines, which were also
inflamed ; aud the witness felt certain that it could not have been
produced otherwise than by the operation of an active poison.?
After this investigation, the witness turned round to the basin,
and was surprised to find it empty: he asked the prisoner what
he had done with the stomach, and he replied that he had thrown
them into the chamber-vase, where was a quantity of water, with
which the intestines had been washed. The witness said, that it
would give him a great deal more trouble in making experiments
to ascertain if any poison were in the stomach, as he must evapo.
rate about two quarts of water. Mr. Street took charge of the
chamber-vase and its contents; it was pushed under the bed. The
witness afterwards applied to it various chymical tests, and they
indicated the presence of arsenic in a fluid, but he could not de-
tect it in a solid state. He had not the slightest doubt that arsenic
was the cause of the death of Mrs. Downing, and he should have
been of that opinion had he not analyzed the contents of the
stomach."
" Dr. Edwards, on being recalled by Mr. Justice Abbot, said,
that 80 parts of cold water would dissolve one part of arsenic ;
that warm water would dissolve a larger proportion, and that then
two or three tea.spoonsfull, or a table-spoonfull, would probably
produce death."
Is it possible not to feel surprised at the boldness of the physi.
cian who should have no doubts on such slender evidence? The
stellated inflammation, as it is called, was probably no more than
blood-vessels denuded by the action of the gastric juice on the
surrounding parts; and the surface of the stomach, softened so as
easily to be scraped off, was the regular process of digestion, as
described by Mr. Hunter, when it takes place after death. Why
did not Dr. Edwards put into a dead stomach some of the solution
of arsenic, of which, he says, a table-spoonful would be enough to
produce death ? or why did he not mix some such solution with the
contents of Mrs. Dunning's stomach, and see how far a corrosion
would have taken place. Corrosion is a chemical process, and should
follow the application of a corrosive substance to dead as well as
living
Medical and Philosophical Intelligence. 431
living animal matter. Inflammation may be excited by a variety
of causes; and, in all sudden deaths with food in the stomach, we
should be prepared to cxpect a partial digestion of the stomachy
Of this we shall say more when our experiments on oxalic acid
are completed.
<c About the middle of January 1815, a young sheep was ob-
served by the shepherd to be unwell, from its heavy and stupid
appearance, such as standing still and bleating, and continually
losing itself, or leaving the Hock to which it belonged; the disease
gradually, but continually, increasing, until it put on the certain
symptoms of water gathering on the brain ; that is, by the animal
constantly inclining to one side, until at last it describes a circle,
which it gradually decreases until it is unable to move more than
the length of its body, From the first appearance of the disease
until its fatal termination is generally from two to three months,
The present subject was taken from a large flock, and put into an
endfosure close by my house; thus giving me an opportunity of
seeing all its actions. One day, being frightened, it ran into a
deep pond, in which it continued swimming in a rotatory manner
until nearly exhausted before it could be got out. I am at present
unable to determine as to the cause of the animal always turning to
the affected side, whether it proceeds from a partial paralysis of
the side affected (as they do not seem to feel the effect of the nerves
crossing) or from the animal constantly leaning its head to the pain*
ful side. The hydatid seldom occupies more than one lobe of the
brain, sometimes posterior, sometimes anterior, is situated beneath
the dura mater, and depresses the substance of the brain; but there
does not appear to be any part of it absorbed. That part of the
cranium immediately over the hydatid is so much reduced by ab-
sorption, that it is found to yield by pressure of the thumb and fin-
ger. Indeed, I have seen it completely absorbed, and the brain
protruding itself between the cranium aud scalp, the animal still
living in that state. This case was operated upon about six weeks
from its first appearance, by turning back the scalp (having first
ascertained the situation of the hydatid by pressure) and applying
a triphine immediately, the bone was removed. The cyst pro-
truded through the orifice, and was taken out by the fingers. A
slight haemorrhage took place, which stopped by gentle pressure.
The wound was then dressed, and healed in about a month. The
animal was afterwards turned into some grass pasture, and appears
going on well. Simple puncture does not answer; for, unless the
cyst is removed, the water accumulates again."?From Mr. Bar-
chard's paper in Annals of Philosophy.
Snake or Adder found in a Block of Coal.?In a recent number
of the Philosophical Magazine, was given a communication on the
singular circumstance of two lizards having been discovered in a
chalk.bed in Suffolk, sixty feet below the surface. The publica.
< - tioo
43$ Medical and Philosophical Intelligence.
tion of this fact has given rise to the following affidavit of a similar
discovery by two pitmen in the county of Stafford. " We, Wil-
liam Mills and John Fisher, both of the parish of Tipton, in the
county of Stafford, do hereby certify and declare, that a few years
ago in working in a certain coalpit belonging to the Right Honor-
able Viscount Dudley and Ward, at what is called the Pieces in tha
parish of Tipton aforesaid, and on cleaving or breaking the stratum
of coal called the stone coal, which is about four feet thick, and in
that situation lies about fifty yards from the earth's surface?we
discovered a living reptile of the snake or adder kind, lying coiled
up, imbedded in a small hollow cell within the said solid coal, which
might be about twenty tons in weight. The reptile when disco-
vered visibly moved, and soon afterwards crept out of the hole;
but did not live longer than ten minutes on being exposed to the air,
when it naturally died, not having been at all hurt by the cleaving
of the coal, whose thickness and solidity must have kept it before
from all air. The hollow in which it lay was split or cloven in two
by means of an iron wedge; and was rather moist at the bottom,
but had no visible water. It was nearly the size of a common tea-
saucer; and the reptile was about nine inches long, of a darkish
ashy color, and a little speckled. After it was dead it was thrown
aside; and the large coal in which it lay, being broken to pieces,
was drawn up out of the pit, and disposed of in the usual way.
In testimony of these facts we have certified the same upon
oath before the Rev. Dr. Booker, a magistrate, this 5th day of
March 1817. Witness our hands,
(Signed) William Mills.
The ><! mark of Joun Fisher.
In the presence of William Summers."
%'* Properly authenticated cases of similar discoveries will al-
ways be recorded with pleasure in our pages; and those who are
alive to the interest excited by such communications arc requested
to communicate them as often as they may come to their know-
ledge.
A Caution.?The following accident happened at Munich on tho
12th of February:?An apothecary's shopman being engaged in
beating up, in a mortar of serpentine stone, a mixture of oxymu-
riate of potash, sulphur, sugar, and cinnabar, for the purpose of
making chemical matches, a terrible explosion took place, which
killed the person who was making the mixture, wounded the apo-
thecary, who at that instant entered, blew the mortar to pieces,
and damaged the stove and furniture of the room.
At a recent sitting of the Society of Medicine of Paris, M.
1'Espagnal communicated a very singular fact:?A workman of a
village at a considerable distance from the capital, in barking the
branches of trees, totally cut off the last phalange of the index fin-
ger of the left hand, and went home; his mother had the idea of
3 replacing
Medical and Philosophical Intelligence. 433
replacing the part cut off, and went and found it amongst the chips
where he had left it, and eighteen minutes after the accident she ap-
plied the parts together, and they united perfectly, but remained in
a state of atrophy for some time, but by slow degrees it recovered
fys sensibility.
Numerous cases have recently been quoted in the various Medi-
cal Journals of Europe, of examples of uniting by a suture, and in
some cases by the first intention; but in, we believe, all these cases
the part was not entirely cut off, but hung by a particle of the skin.
This case, well authenticated, proves that this is not absolutely ne-
cessary to insure success.
Dr. Husson, who had so greatly contributed to the propagation
of vaccination in France, found recently, on opening the body of a
woman at the Hotel Dieu, a polypusin the stomach ; it measured
eight inches in length, and six-tenths of an inch in diameter; this
body, which is of a greater size than any hitherto discovered, ad?
hered to the membrane of the stomach, but it did not appear to
have had any action in the vital functions, nor had it any influence
in the cause of her death. Dr. Brechet has made a drawing and
preparation, with a description, of this polypus; and we hope next
month to give his Memoir in the Medical and Physical Journal.
A suture of the membrane of the stomach has been the subject
of a memoir read at the Faculty of Medicine, by the Baron Percy.
The success with which this operation has been performed in more
than one case, has removed all apprehensions of these wounds being
invariably mortal, as they had previously been considered.
The Journal Universel des Sciences Medicaids contains a propo-
sal for the translation of Garth's Dispensary. The compilers give
a general analysis of the work, which they say, " donncra une idee
du genre de plaisanterie que les Anglais appellent humour. Cet
ouvrage, traduit en vers, paraitrait fort piquant, si Ton en juge par
1'exorde que Voltaire a rime :
Muse, raconte.moi les debats salutaires
Des medecins de Londre et des apothicaires.
Contrele genre humain si long-tems reunis,
Quel dieu, pour nous sauver, les rendit ennemis ?
Comment laisserent-ils respirer leurs malades .
Pourfrapper ?t grands coups sur leurs chers camarades?
Comment changerent-ils leur coiffure en armet,
La seringue en canon, la pilule en boulet ?
lis connurent la gloire ; acharnes l'un sur l'autre,
Us prodiguaieht leur vie et respectaicnt la notre."
We very much fear this inimitable poem would lose much of its
force in a French dress, and even in the meridian of France, where
the distribution of the medical offices is so different; the apotheca-
ries never visiting, but confining themselves to compounding at
u So.21^ &kv home.
434 Medical and Philosophical Intelligence.
home. The present custom in London seems to have been recent
even in Garth's time, and to have led to the proposed dispensary at
the college, that those who could not afford to fee physicians might
jiave advice gratis from that order of the profession, and, by re-
ceiving the medicine gratis, the prescriptions might not fall into
the hands of apothecaries, who, at that time, had no medical
knowledge from any other source.
" Thus, modern apothecaries taught the art,
IJy doctor's bills to play the doctor's part, &c." Garth.
We cannot but approve the modern custom of requiring a suit-
able education, and, at length, an examination of men whom the
public will consult, and who cannot be expected to withhold their
gdvice.
On the Venom of the Viper. Extracted from the Discorso del Sig.
Prof. Mangili, intorno al Veleno della Vepera, letto al R. I.
< Instituto.
The ancients were of opinion that the poison of vipers, intro.
cluced direct into the alimentary canal, was not dangerous. They
founded their argument on the fact, that one might with impunity
suck the wound made by a viper, carefully spitting it out; and
w hich was one of their remedies. Redi has adopted this opinion.
Since that period, Fontana advanced that, if a small dose of
poison might be taken by man without danger, on account of
Ins bulk compared with that of the viper, a larger dose would bo
dangerous, and even fatal. He cut off the heads of eight vipers,
and expressed all the venom into a tea-spoon, and introduced it
into the stomach of a pigeon, which had had no food for eight
hours. In less than a minute the animal appeared weak, in two
minutes more it began to stagger, fell on its side, and died in six
minutes in strong convulsions.
This experiment is the reverse of that of Redi, who, having di-
luted the poison of four vipers in a glass of water, and having
given part to a goat and part to a duck, perceived no effects re-
sulting from it.
At length, Jacob Strozzi drank with impunity the poison of a
?viper diluted in half a glass of wine: at another time he drank the
poison of three vipers, diluted in a similar manner.
Wishing to clear up the point, M. Mangili took four small
blackbirds. The first drank the fluid venom of three vipers, the
second that of four, the third that of five, and the fourth that of
six, of these animals. They appeared at first plunged into a state
of stupidity and inaction, but in less than an hour they were as
lively as before, and ate with appetite.
He then gave the venom of upwards of twenty vipers to a little
puppy, in which it produced no effect.
These experiments were so conclusive, that a person present
swallowed the venom of four large vipers without being affected
by it.
? I Th?
Medical and Philosophical Intelligence, 435
the following year the experiment was repeated on a crow,
which had fasted twelve hours: it drank with impunity the venom
of sixteen vipers.
In October, 1814, says the author, I forced seven large vipers
to shed all their venom into a cup. I steeped in it crumbs of
bread, and made a pigeon swallow them. At first it appeared ill,
but soon recovered itself. Some days afterwards t introduced into
its food, as well as that of another pigeon, a small quantity of
venom, very dry, which had been kept fourteen months in a glass
hermetically closed. They both manifested signs of being poi-
soned, and died in about two hours.
Another pigeon swallowed the poison of ten large vipers with-
out it at all affecting it.
Fontana asserted that dry poison lost its virtues in nine months.
The above experiment shews the contrary. In introducing poison
into a wound, I cover the wound with court plaster, in order that
the poison may not be rejected by the flowing of the blood.
I have made similar experiments on pigeons with poison kept
eighteen, twenty-two, and even twenty-six months. The effcct
was always the same; and therefore I have no hesitation in con-
tradicting the assertion of Fontana, and to affirm, that poison,
carefully kept, will retain its noxious qualities for years.
The situation of the Duke of Newcastle's family at Clumber
Lodge is an object of most important inquiry. Besides his Grace,
fifteen of the family, including servants, are ill of fevers. The
Duchess has been delivered of a dead child, but whether from
a slighter attack of the fever, or anxiety on account of the Duke,
Is not ascertained. In the present uncertainty, the cause is va-
riously surmised. The most current report is, that a lake being
dried, and the soil removed for the purpose of manure, its effluvia
produced this dangerous miasma. This is the more remarkable in
bo cold a season. We have heard that, in some parts of the country
which have been drained near the banks of the Ouse, continued
fever has taken place of the former intermittents. We shall be
thankful to any practitioner in these parts, or near the Duke's
neighbourhood, who will favour us with information.
In the course of next month will be published in London, No. 1,
of the Continental Medical Repertory, exhibiting a concise view
of the latest discoveries and improvements made on the continent in
Medicine, Surgery, and Pharmacy. Conducted by E. Von Lmb-
den, M.D. assisted by other gentlemen of the faculty.
In the press, and will speedily be published, an Experimental
Inquiry into the Laws of the Vital Functions, with some Observa-
tions on the Nature and Treatment of Internal Diseases. By A.
P. Wilson Philip, M.D. In part re-published from the Philoso.
phical Transactionsj with the Report of the National Institute of
& k 2 France
43fi Report oj Diseases.
France on the Experiments of M. Ie Gallois, and Observations on
that Report. ?
In the press, the Consulting Surgeon, or Practical Essays, Cases,
and Observations, illustrating the more important Surgical Diseases.
By John Bell, Edinburgh. The object of this work is to represent,
in regular series, 1st, the influence of education on the talents,
manners, and conduct, of the medical profession ; 2dly, the duties
of consulting and operating surgeons ; Sdly, the influence of inerted
vascular action in ruining the organization of its several parts and
organs of the animal body; 4thly, Consultations, Narratives, and
Descriptions, of the more important injuries or diseases of the
limbs, of the skull, of the brain, of the arteries, of the urethra and
perineum, and of the urinary bladder. With sketches of the seve-
ral parts in their natural and in their diseased condition ; with
plans of the necessary operations, in fifty engravings.
Dr. Davis will commence his first Summer Course of Lectures
on the Theory and Practice of Midwifery, and on the Diseases of
Women and Children, on the 20th of May.
Dr. Clutterbuck. will begin his Summer Course of Lectures on
the Theory and Practice of Physic, Materia Medica, and Chemis-
try ; on Monday, June the 2d, at ten o'clock in the morning.
Theatre of Anatomy, Hatton Garden.?Mr. Taunton's Sum-
mer Course of Lectures on Anatomy, Physiology, Pathology, and
Surgery, will commence on Saturday, May 24th, ]817, at eight
o'clock in the evening precisely, and be continued every Tuesday,
Thursday, and Saturday, at the same hour.
REPORT OF DISEASES. '
The continued coldness of the weather produces a continuancfe
of inflammatory diseases, chiefly pulmonary. Many subjects who
appeared convalescent and likely to pass the summer without in-
convenience have relapsed, and are threatened with phthisis, unless
the season should become more genial.
Typhus is said to be prevalent, which may be expected from the
continuance of cold, added to the other distresses of the poor.
Hitherto, however, the benevolence of the higher orders has pre-
vented that abject distress, which, under similar circumstances on
former occasions, produced pestilence in addition to famine.
Some cases of small-pox after vaccination have occurred, which
appeared at first alarming, but in the end proved of the mitigated
kind; they have all dried early, and we doubt not the uneasiness
they have excited will gradually subside.
Scarlatina has been very prevalent, but rarely fatal,
[We
Meteorological Register for February. 437
[We have much regretted the disappointment experienced by
the various engagements of our former Reporter, in consequence
of which the Register has been o:nitted in the two preceding
Numbers, To render tho work a complete rccord in this as in
every tiling else connected with medicine, we now continue the
Register from the date of our last,]
A METEOROLOGICAL JOURNAL,
By Messrs. William IIaiuus and Co. 50, Uolborn, London.
From the 20tk January to the 1 Qth February, inclusive.
Day
of
iMontli
Rail
gauge
Jan.
20
21
2 2
23
24
25
26
2f
28
29
30
31
Feb.
1
2
3
4
5
6
7
8
9
10
11
12
13
14
15
16
17
18
19
05
03
,03
,12
,19
,10
.23
rHERM.
Barom
10 49
49
46
45
28?129
29 9
45
50
51
51
51
49
38
41
50
47
48
46
49
49
47 44
29s
298
298
30
303
30 2
304
304
303
303
304
305
304
303
30
29 8
30
0
44130 3
304
302
299
299
30
29s
r)S
29 s
30
30
302
29'
29 9
30
303
30a
304
303
30
303
30s
305
04
30
29 6
30
301
30
302
303
30
30
30
29
29
295
30
30
30
301
Da Luc's
IIygrom.
Dry. L>ii1111?
Wind.
SSE
sw
sw
SW
w
sw
ssw
sw
E
sw
vv
NW
NNW
VV
sw
sw
w
wsw
wsw
w?w
. sw
W va.
NNE
W
S
w
wsw
sw
sw
sw
w
sw
ssw
sw
w
sw
ssw
ssw
wsw
sw
w
NW
NW
NNW
WSW
ssw
ssw
ssw
wsw
w
sw
ssw
sw
sw
w
sw
sw
w
sw
sw
sw
sw
Atmospheric
Variation.
i* I IVtr
Fine
Clo.,
Clo.
Fin<-
Clo.
,:i?.
Jio.
Clo.
Fine
Fine
Fine
Fine
Fine
Clo.
Fine
Fine
Clo.
Fine
Fine
Fine
Clo.
Rain
Fine
Rain
Fine
Rain
Fine
Clo.
Clo.
Fine
Clo.
Fine
Rain
Fine
Fine
Rain
Fine
Fine
Fine
Fine
Fine
Fine
Fine
Rain
Fine
Clo.
.Clo,
Clo.
Rain
Ran,
The quantity of rain fallen in the month of January was
2 inches and 68-100ths,
A Mcteo-
438
A METEOROLOGICAL JOURNAL,
By Messrs. William Harris and Co. 50f Holborn, LonJuw,
From the 20th February to the 19th March, inclusive.
Day
of
Month.
Feb.
20
21
22
23
24
25
26
27
28
March
1
2
3
4
5
6
7
8
9
10
11
12
13
14
15
16
17
18
19
Rain
gauge
,19
,12
,08
,10
04
,06
,14
,10
,11
,10
,18
,43
fllERM
Barom
296
29 6
29 6
297
296
30 ,
301
29 6
299
29?
29s
294
29
293
292
293
291
29 7
30
302
30
30
302
30 3
302
30 3
303
29 9
295
295
296
297
30
30
30
29 9
299
29?
292
29
292
293
292
293
294
29"
302
30
299
30
30 3
303
302
303
30?
29?
De Luc's
Hygrom.
Dry. Ddiiijj
Wind.
SW
W
SW
NNW
w
ssw
w
VV va.
WSW
W
SW
ssw
W va.
SW
WNW
SW
w
w
\\
SW
s
WSW
NE
SE
E
NE
SE
SW
WSW
w
w
NW
SW
SW
VV vn.
SSW
w
SW
SW
SW
W va.
SSW
SW
SSW
WSW
w
SW
ssw
SW
WNW
E
s
E
NW
SW
WSW
Atmospheric
Variation,
Clo,
Clo.
Clo.
Fine,
Fine
Fine
Rain
Fine
Clo.
Clo.
Fine
Fine
Clo.
Fine
Rain
Fine
Sleet &
Rain
Fine
Fine
Fog
Fine
Fine
Fine
Fog
Clo.
Fog
Fog
Clo.
Rain
showery shower.
Clo.
Clo.
Fine
Fine
Fine
Fine
shower,
shower,
shower.
Fine
Fine
Clo.
Clo.
Fine
Fine
Fine
shower,
Clo.
Clo.
Clo.
Clo.
Fine
Fine
Fine
Fine
Rain
Rain
Rain
Rain
Rain
Clo.
Fine
Clo.
Fine
Clo.
Fine
Clo.
quantity of rain fallen in the month of February is
1 inch and 26-lOOths.
A Meteoro-
439
A METEOROLOGICAL JOURNAL,
By Messrs. William Harris and Co. 50, Holborn, London*
From the QOth March to the 19th April, inclusive.
Day
of
Month.
Rain
gauge
Marcl
20
21
22
23
24
25
26
27
28
29
30
31
April
1
2
3
4
< 5
6
7
8
9
10
11
12
13
14
15
16
17
18
19
Therm
03
03
Barom,
55
54
55
53
47
45
47
48
47
40
43
49
49
55
58
47',49
1349
4349
4553
298
29?
299
30
299
298
30
301
30
299
30i
304
44
306
30s
304
304
304
304
305
30'
30
301
303
301
30=
301
30
301
303
38 304
30s
299
299
30
30
299
29
29?
302
30
299
301
306
305
304
304
304
304
305
304
30
301
30 3
30
30
30:
30
29
30
30
30s
304
De Luc's
Hyghom.
Dry Damp
Wind.
W
NW
N
SE
S
SSW
S
NNW
S
s
s
SSW
SSE
ENE
NE
NNE
NE
NE
ENE
SE
WSW
NW
NTNW
NW
WNW
W
w
MNW
N
NNE
N va.
NNW
NNW
NE
S
SSW
SF
WNW
WSW
SSW
SW
SSW
SE
ESE
ENE
NE
NNE
E *
NE
ENE
SW
W
NW
NW
W
w
W
NW
N va
N
W
N va.
A'mospheric
Variation.
Fine
Fine
Fine
Fine
Fin-
[tain
Clo.
Fine
Clo.
Clo.
Fine
Fine
Fine
Fog
Fint
Fine
lrint
Fine
Fint
Fog
Fog
Fine
Fine
Fine
Clo.
Fine
Fine
(lain
Fine
Clo.
Fine
Snov>f CMo.
Clo.
Clo.
Clo.
Fine Clo.
Fine
Fine
Clo.
Fine Clo.
Fine
Rain Clo.
Clo.
The quantity of rain fallen in the month of March is
1 inch and 12-100ths.
To

				

